# SARS-CoV-2 surveillance and detection in wild, captive, and domesticated animals in Nebraska: 2021–2023

**DOI:** 10.3389/fvets.2024.1496207

**Published:** 2025-01-03

**Authors:** Duan Sriyotee Loy, Rachael Birn, Korakrit Poonsuk, Bryan Tegomoh, Amanda Bartling, Michael R. Wiley, John Dustin Loy

**Affiliations:** ^1^School of Veterinary Medicine and Biomedical Sciences, Nebraska Veterinary Diagnostic Center, University of Nebraska-Lincoln, Lincoln, NE, United States; ^2^Department of Environmental, Agricultural and Occupational Health, University of Nebraska Medical Center, Omaha, NE, United States; ^3^Washington Animal Disease Diagnostic Laboratory, Department of Veterinary Microbiology and Pathology, College of Veterinary Medicine, Washington State University, Pullman, WA, United States; ^4^Division of Public Health, Nebraska Department of Health and Human Services, Lincoln, NE, United States; ^5^Department of Pathology, Microbiology, and Immunology, College of Medicine, University of Nebraska Medical Center, Omaha, NE, United States

**Keywords:** SARS-CoV-2, surveillance, deer, cat, wildlife, captive

## Abstract

Widespread surveillance for SARS-CoV-2 was conducted across wildlife, captive animals in zoological collections, and domestic cats in Nebraska from 2021 to 2023. The goal of this effort was to determine the prevalence, phylogenetic and spatial distribution characteristics of circulating SARS-CoV-2 variants using various diagnostic methodologies that can utilize both antemortem and postmortem samples, which may be required for wildlife such as white-tailed deer. Statewide surveillance testing revealed high variation in SARS-CoV-2 prevalence among species, with white-tailed deer identified as the primary reservoir. In 2021, seroprevalence in white-tailed deer was 63.73% (*n* = 91) and 39.66% (*n* = 237) in 2022, while virus detection in retropharyngeal lymph nodes (RLN) was 16.35% (*n* = 483) in 2021 and 3.61% (*n* = 277) in 2022. Phylogenetic analysis was conducted on 11 positive samples from 2021. This analysis revealed the presence of four lineages of the Delta variant: AY.100, AY.119, AY.3, and AY.46.4. Conversely, other species showed no virus detection, except domestic cats, which had a low seroprevalence of 2.38% (*n* = 628) in 2022, indicating minimal exposure. The detection of SARS-CoV-2 in white-tailed deer and the identification of multiple Delta lineages underscores the need for ongoing surveillance and the importance of using different diagnostic methodologies. These efforts are critical for understanding virus circulation and evolution in wildlife and domestic animals, informing public health strategies, and mitigating the risks of zoonotic transmission of SARS-CoV-2 and other emerging infectious diseases.

## 1 Introduction

Severe acute respiratory syndrome coronavirus 2 (SARS-CoV-2), the causative agent of coronavirus disease 2019 (COVID-19), emerged in late 2019 and rapidly spread globally, causing a pandemic (1, 2). SARS-CoV-2 is believed to have originated from bats and made the zoonotic jump into humans. However, there is evidence that the virus can also infect a wide range of animal species. Studies have detected SARS-CoV-2 infections in domestic and wild animals, including; felines, canines, mink, cervids, primates, and rodents (3–11) highlighting the potential for diverse animal reservoirs to potentially influence virus persistence and evolution. The subsequent emergence of numerous SARS-CoV-2 variants, classified by the World Health Organization (WHO) as variants of concern (VOCs) and variants of interest (VOIs), has revealed significant differences in transmissibility, disease severity, and immune escape capabilities, emphasizing the critical role of genomic surveillance in guiding public health strategies (12).

Among these reservoirs, white-tailed deer (*Odocoileus virginianus*) have been identified as a reservoir species of SARS-CoV-2 with both seroprevalence data and RT-PCR detections indicating widespread infection in populations across several states (13–21). Similarly, domestic cats have shown susceptibility to the virus, to which exposure is thought to be primarily derived through human contact (5, 22–29). Understanding the distribution and evolutionary dynamics of SARS-CoV-2 in deer, cats, and other animal populations is important for understanding potential human-animal transmission and monitoring the emergence of novel variants and ensuring the health of a variety of animal species. Given the diverse clinical presentations and varying degrees of susceptibility among these species, which may include asymptomatic infections, we aimed to assess the degree of virus circulation as broadly as possible. In this study, we conducted a statewide surveillance study to determine SARS-CoV-2 prevalence in wildlife, captive animals in zoological collections, and domestic cats in Nebraska over a three-year period (2021–2023), with the objectives to determine the prevalence and phylogenetic characteristics of circulating SARS-CoV-2 variants using different diagnostic methodologies that enable both postmortem and antemortem sample collection and analyze the spatial and phylogenetic characteristics of detected variants.

## 2 Materials and methods

### 2.1 Study population and sampling

**Figure 1:**
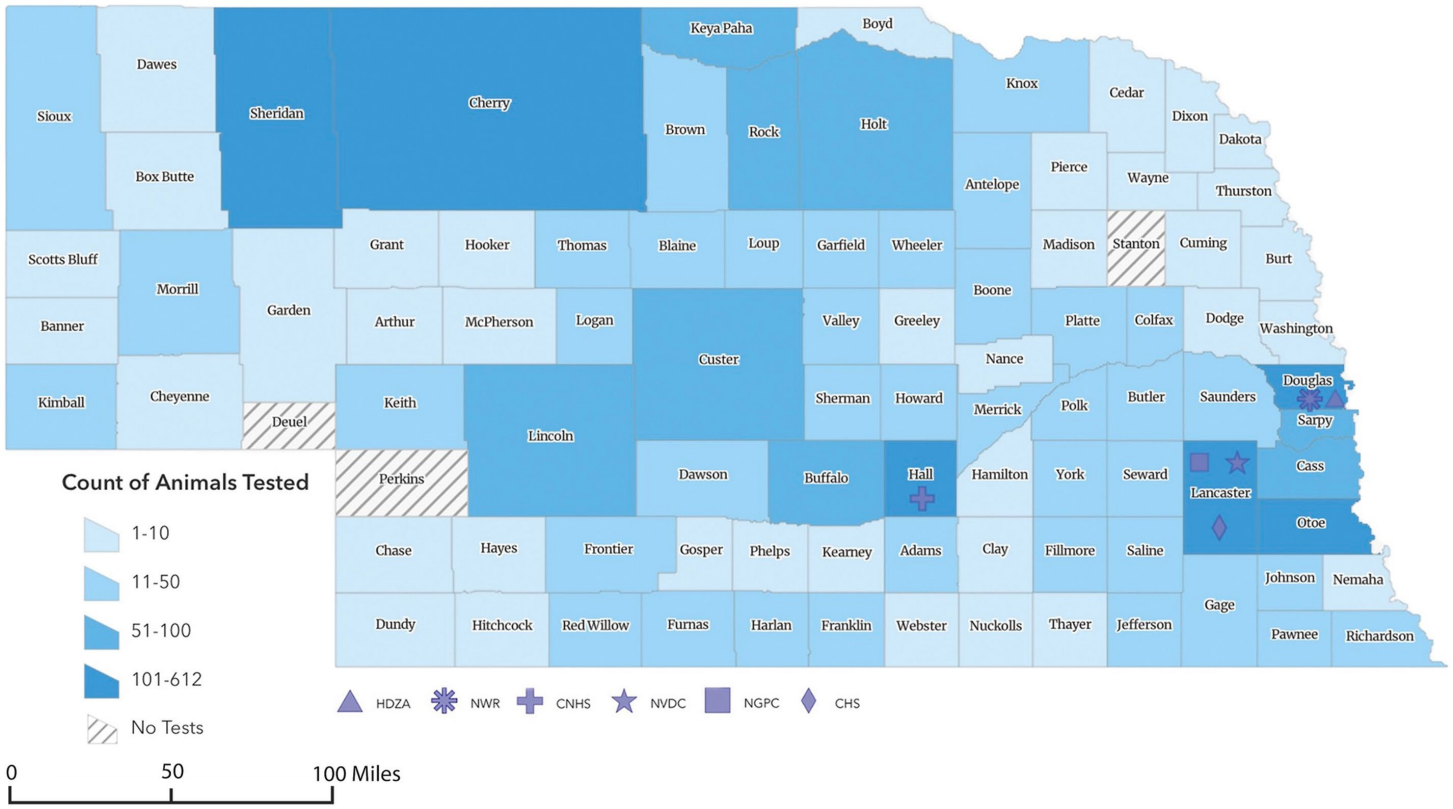
Geographic choropleth map of animals sampled for SARS-CoV-2 surveillance program in Nebraska from 2021 to 2023. The color intensity represents the relative number of animals collected from each county (*n* = 3,631). *Counties sampled represented 90 out of the 93 counties in Nebraska. **Of 3,631 total samples collected, the location of 146 was unknown and 4 were collected out of state (two from Iowa and one each from Kansas and Minnesota). The symbols representing HDZA, NWR, CNHS, NVDC, NGPC, or CHS indicate location of primary clinic, main office, or laboratory and not necessarily the origin of samples collected by those agencies.

To conduct this study, a partnership among Henry Doorly Zoo and Aquarium (HDZA), Nebraska Game and Parks Commission (NGPC), Capital Humane Society (CHS), Central Nebraska Humane Society (CNHS), Nebraska Wildlife Rehab (NWR), Nebraska Veterinary Diagnostic Center (NVDC), and Nebraska Department of Health and Human Services (NDHHS) was established as part of the statewide SARS-CoV-2 virus surveillance program. The objective of this collaboration was to determine the prevalence and phylogenetic characteristics of circulating SARS-CoV-2 variants in diverse potential host species across Nebraska. Through these collaborations, 5,794 samples were collected from various locations by collaborated organizations (Figure 1). Samples were obtained from a diverse array of 3,631 animals, representing 44 species (Table 1), including zoo animals, peri-domestic feral cats, wildlife, and animals submitted to the NVDC for rabies testing as part of a statewide rabies surveillance program. The surveillance sampling strategy involved acquiring respiratory, feces, lymph nodes (deer and cervids), and blood samples when feasible. Sample types collected varied depending on the location, management practices, and the accessibility of different species for sampling (Table 2). Stringent safety measures were implemented during sample collection, with collectors equipped with appropriate personal protective gear such as gloves, face masks, and disposable laboratory coats. The data including the animal’s location of identification, sex, age, and any observable symptoms were recorded. However, the information varied based on the classification of animals and their known histories, with variations depending on the source, e.g., zoological collections, captured wildlife, or domestic animals.

**Table 1:** The table presents data from the SARS-CoV-2 surveillance program in Nebraska, spanning from 2021 to 2023.

Species	Breed	Scientific name	Number unique animals	Number of samples	Environment/animal location
Deer	Mule	*Odocoileus hemionus*	261	420	Wildlife
	White-Tailed	*Odocoileus virginianus*	1,378	1,883	Wildlife
Domestic cat	Domestic Longhair	*Felis catus*	958	1,251	Domestic/Peri-domestic
Non-human primate	Black and white ruffed lemur	*Varecia variegata*	7	152	Zoological Collection
	Crowned lemur	*Eulemur coronatus*	2	39	Zoological Collection
	Gorilla	*Gorilla gorilla*	12	252	Zoological Collection
	Orangutan	*Pongo pygmaeus*	4	90	Zoological Collection
	Red Ruffed Lemur	*Varecia rubra*	8	178	Zoological Collection
	Siamang Gibbon	*Symphalangus syndactylus*	3	66	Zoological Collection
	Squirrel monkey	*Saimiri sciureus*	6	151	Zoological Collection
Bat	Egyptian Fruit Bat	*Rousettus aegyptiacus*	330	330	Zoological Collection
	Big brown bat	*Eptesicus fuscus*	295	297	Wildlife
	Hoary bat	*Lasiurus cinereus*	4	4	Wildlife
	Silver-haired bat	*Lasionycteris noctivagans*	1	1	Wildlife
Large cat	African Wildcat	*Felis lybica*	1	19	Zoological Collection
	Amur Tiger	*Panthera tigris altaica*	3	53	Zoological Collection
	Bobcat	*Lynx rufus*	1	19	Zoological Collection
	Cheetah	*Acinonyx jubatus*	2	40	Zoological Collection
	Snow Leopard	*Panthera uncia*	2	52	Zoological Collection
Coati & raccoon	Raccoon	*Procyon lotor*	73	73	Wildlife
	White-nosed Coati	*Nasua narica*	1	19	Zoological Collection
Mink/Otter	Asian small-clawed otter	*Aonyx cinereus*	2	40	Zoological Collection
	Mink	*Neovison vison*	1	1	Wildlife
	Spotted-neck otter	*Hydrictis maculicollis*	2	42	Zoological Collection
Elk	Elk	*Cervus canadensis*	72	72	Wildlife
Rodent	13 Lined Ground Squirrel	*Ictidomys tridecemlineatus*	1	1	Wildlife
	American Porcupine	*Erethizon dorsatum*	1	1	Wildlife
	E Gray Squirrel	*Sciurus carolinensis*	1	1	Wildlife
	Eastern fox squirrel	*Sciurus niger*	35	36	Wildlife
	Ground hog/Woodchuck	*Marmota monax*	12	12	Wildlife
	Muskrat	*Ondatra zibethicus*	1	1	Wildlife
	North American Beaver	*Castor canadensis*	3	3	Wildlife
	Rat	*Rattus norvegicus*	1	1	Wildlife
Opossum	Virginia Opossum	*Didelphis virginiana*	49	49	Wildlife
Fossa	Fossa	*Cryptoprocta ferox*	2	48	Zoological Collection
Rabbit	Eastern Cottontail	*Sylvilagus floridanus*	36	36	Wildlife
Pronghorn	Pronghorn	*Antilocapra americana*	32	32	Wildlife
Dog	Domestic Dog	*Canis familiaris*	10	11	Domestic
Skunk	Skunk	*Mephitis mephitis*	7	7	Wildlife
Armadillo	Armadillo	*Dasypodidae*	2	2	Wildlife
Bovine	Bovine	*Bos taurus*	1	1	Wildlife
Bird	Robin	*Turdus migratorius*	1	1	Wildlife
Coyote	Coyote	*Canis latrans*	4	4	Wildlife
Fox	Fox	*Vulpes vulpes*	3	3	Wildlife
Grand total			3,631	5,794	

It categorizes the animals tested by species and breed, detailing the count of unique individuals and the total number of samples collected.

**Table 2:** Sample type tested (Respiratory swabs, feces, retropharyngeal lymph node (RLN) Nobuto strips, serum, or RLN exudates), year of collection (2021, 2022, or 2023), location/collaborator source (Henry Doorly Zoo and Aquarium (HDZA)), Nebraska Game and Parks Commission (NGPC), Capital Humane Society (CHS), Central Nebraska Humane Society (CNHS), Nebraska Wildlife Rehab (NWR), Nebraska Veterinary Diagnostic Center (NVDC), and Nebraska Department of Health and Human Services (Others), and testing method (Real Time PCR (RT-PCR) or surrogate virus neutralization test (sVNT)) used for the study.

Locations	Total samples	Sample types														
		RT-PCR									sVNT					
		Respiratory swabs		Feces		RLN			Nobuto strips		Nobuto strips		Serum		RLN Exudates	
		2022	2023	2022	2023	2021	2022	2023	2022	2023	2022	2023	2022	2023	2022	2023
HDZA	1,590	0	0	1,047	543	0	0	0	0	0	0	0	0	0	0	0
NGPC	2,409	625	0	0	0	483	277	606	105	0	76	0	0	0	237	0
CHS	947	379	16	0	0	0	0	0	0	0	0	0	311	241	0	0
CNHS	236	118	42	0	0	0	0	0	0	0	0	0	0	76	0	0
NWR	134	0	0	112	22	0	0	0	0	0	0	0	0	0	0	0
NVDC	475	468	7	0	0	0	0	0	0	0	0	0	0	0	0	0
Others	3	0	0	3	0	0	0	0	0	0	0	0	0	0	0	0
Totals	5,794	1,590	65	1,162	565	483	277	606	105	0	76	0	311	317	237	0

### 2.2 Sample origins and collection

**Figure 2:**
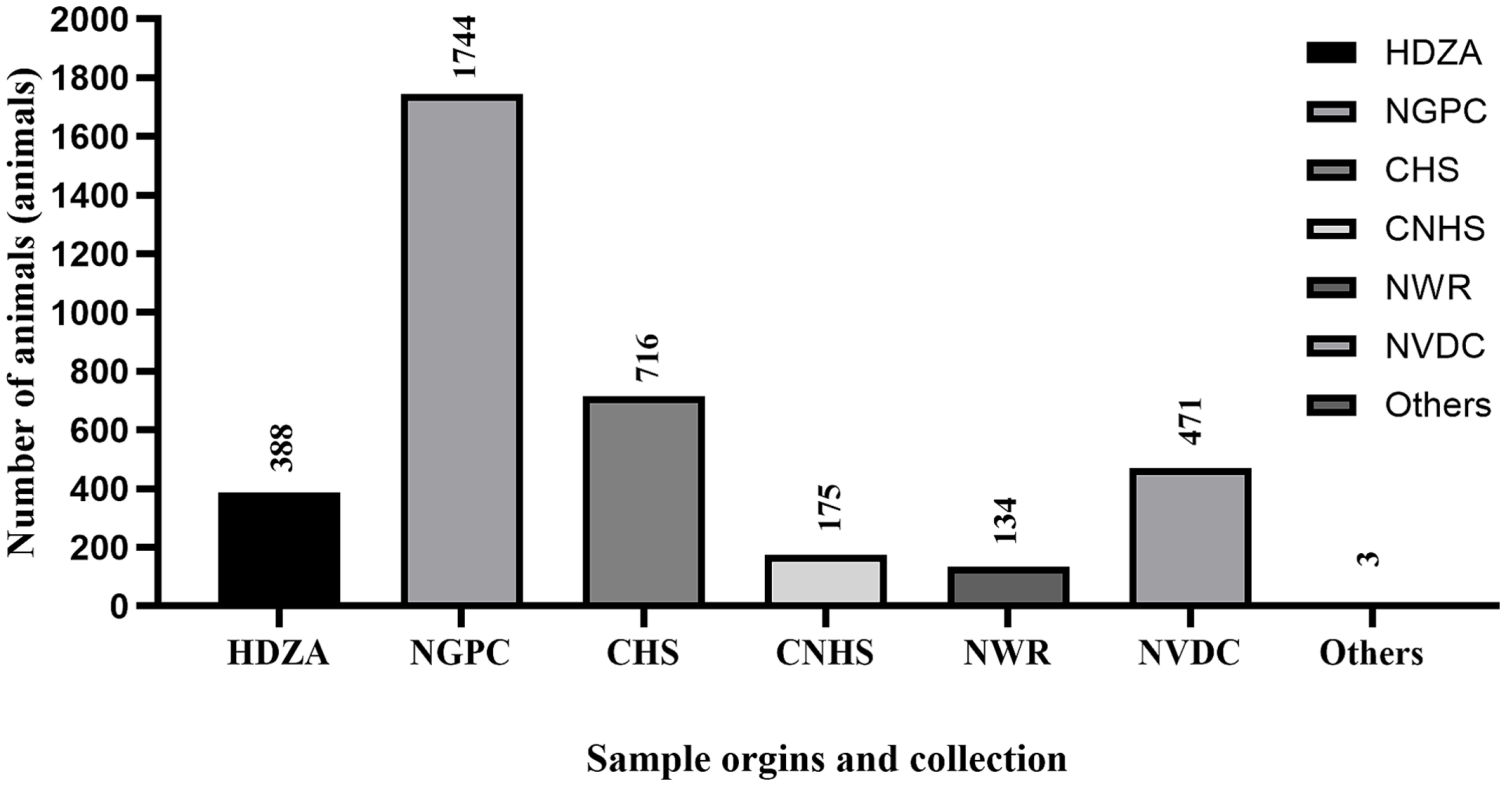
The figure shows the total number of samples (*n* = 5,794) collected from each location including Henry Doorly Zoo and Aquarium (HDZA), Nebraska Game and Parks Commission (NGPC), Capital Humane Society (CHS), Central Nebraska Humane Society (CNHS), Nebraska Wildlife Rehab (NWR), Nebraska Veterinary Diagnostic Center (NVDC) and others.

Specimens included in this study were obtained from various locations throughout Nebraska as part of surveillance programs conducted by different sectors, including the Henry Doorly Zoo and Aquarium (HDZA), Nebraska Game and Parks Commission (NGPC), Capital Humane Society (CHS), Central Nebraska Humane Society (CNHS), Nebraska Wildlife Rehab (NWR), public submissions, and specimens submitted to the Nebraska Veterinary Diagnostic Center (NVDC) for general diagnostics (Table 2; Figure 2). All samples collected were stored at −80°C until testing. Samples represented cross-sectional samplings across these populations, as they are not captive or tracked, except for HDZA, which was serially conducted in captive populations.

#### 2.2.1 Henry Doorly Zoo and Aquarium

Bat guano samples (*n* = 330) were collected bi-weekly from a population of over 1,700 Egyptian fruit bats in a grouped-housed, confined environment at the Omaha’s Henry Doorly Zoo and Aquarium. Bat guano samples were swabbed and placed into molecular transport media (MTM, Longhorn Vaccines and Diagnostics LLC). Fecal samples (*n* = 1,260) were collected bi-weekly from animals of SARS-CoV-2 susceptible species (*n* = 58) as part of the surveillance (Table 1) representing serial sampling from these populations. Staff collected fecal swabs, placed into a tube with viral transport media. Samples were transported to the NVDC by NDHHS staff or shipped overnight on ice packs to NVDC and were frozen at −80°C until testing.

#### 2.2.2 Nebraska Game and Parks Commission

The Nebraska Game and Parks Commission (NGPC) conducted a statewide chronic wasting disease (CWD) surveillance program during the firearm seasons in November 2021, 2022, and 2023, which had been archived and stored at −80°C after collection. As part of this program, retropharyngeal lymph node (RLN) samples were collected from white-tailed deer and mule deer. These samples were submitted to the NVDC for CWD testing and subsequently used as part of this SARS-CoV-2 surveillance study. In 2021, 1,464 RLNs were collected from 6 deer management units in the south central and southeast regions of the state and hunter’s consent allowed the testing of 531 samples, however, only 483 RLNs were available for inclusion in this study. During the 2022 firearm hunting season, multiple partnerships led to sample collection from hunter-harvested deer at 10 deer management units across Nebraska. Hunters provided information including harvested date, age, sex, species, and harvest location. The specimens were obtained with hunter approval. Sampling involved the removal of RLN, along with swabs from nasal, oral, or tracheal cavities, and blood absorption on Nobuto strips when feasible. A total of 519 respiratory swabs, 277 RLNs, 237 serum samples, and 105 Nobuto strips were collected from 550 deer carcasses. In 2023, 606 RLN samples collected in the northern part of Nebraska were included in this study. The county’s location where the RLN samples were obtained is shown in Supplementary Table S1.

#### 2.2.3 Humane societies

As part of an existing Trap-Neuter-Release (TNR) program for peri-domestic feral cats conducted by the Capital Humane Society (CHS) and Central Nebraska Humane Society (CNHS) in 2022 and 2023, swabs (*n* = 555) and blood samples (*n* = 628) were collected from a total of 958 cats by CHS or CNHS staff for SARS-CoV-2 testing. Samples were collected during the cats’ sedation for other surgical procedures. Swabs were gently taken from the nasal or oral cavities using the Minitip Flocked Eswabs^®^ 481C (Copan Diagnostic Inc., Brescia, Italy) collection system, ensuring that the swab tip was saturated with mucus or contained mucosal cells. Whole blood samples were collected into EDTA collection tubes (Becton Dickinson and Company, New Jersey, United States) using aseptic techniques. The samples were then refrigerated and submitted to the NVDC for further testing.

#### 2.2.4 Nebraska Wildlife Rehab

The Nebraska Wildlife Rehab (NWR) focuses on rehabilitating native Nebraska wildlife. Fecal samples (*n* = 134) were collected from newly arrived wildlife in veterinary cages upon intake. NWR staff collected the samples into molecular transport media (MTM, Longhorn Vaccines and Diagnostics LLC). The collected samples were then refrigerated and shipped to NVDC for SAR-CoV-2 RT-PCR testing.

#### 2.2.5 Nebraska Veterinary Diagnostic Center

Between March 2022 and February 2023, NVDC collected nasal swab samples (*n* = 475) from various animals submitted for rabies virus testing, either due to human or non-human exposure, submitted by diagnostic clientele or as part of the statewide rabies surveillance program.

### 2.3 Sample processing

Five nasal swabs or five fecal samples were pooled in equal amounts (per weight or volume) for SARS-CoV-2 RT-PCR testing. Samples were initially tested in pools of five to maximize test capacity. If a pool is positive, the 5 samples were then tested individually to confirm detection status. For each RLN, samples were processed and subjected to high-volume magnetic bead total nucleic acid extraction as previously described elsewhere (18). The remaining lymph node tissues then underwent two freeze–thaw cycles at −80°C, 2 h of freezing per cycle. Exudate that accumulated within the sample bag during the process was transferred to a cryogenic tube for serological testing.

For Nobuto strips, the blood-absorbed portion of each strip was cut into approximately 2×2 mm fragments using sterile surgical scissors, placed onto a weight paper and then transferred to a sterile microcentrifuge tube. Subsequently, 400 μL of dilution buffer from the SARS-CoV-2 surrogate virus neutralization test (sVNT) kit (GenScript; New Jersey, United States) was added to each tube for filter paper saturation. The tubes were then incubated overnight at 4°C to extract serum from the filter paper. After incubation, the tubes were mixed by vortex to homogenize the serum extract. The resulting serum extract was used directly, without further dilution, in the subsequent sVNT enzyme-linked immunosorbent assay (ELISA).

### 2.4 RT-PCR testing and sequencing procedure

SARS-CoV-2 RT-PCR protocol detecting N1 and N2 targets has been previously described in Poonsuk et al. (18). From the subset of 79 positive deer samples in 2021, 20 samples exhibited cycle threshold (Ct) values below 30, indicating higher viral loads were selected for a more comprehensive investigation at the Nebraska Public Health Laboratory (NPHL) for whole genome sequencing (Table 3). In 2022, 10 samples with Ct <30 were submitted for sequencing but all samples failed to produce SARS-CoV-2 specific amplicons (samples not listed).

**Table 3:** Sequencing metadata for the 20 SARS-CoV-2 positive samples processed for whole genome sequencing.

Sample	Host	Collection date	Collection location	Ct value	Raw reads	Sequencing coverage	Assembly coverage	GISAID accession ID	Pango lineage
S513849	*Odocoileus virginianus*	11/13/2021	YORK	26.82	16,881	609x	81.53%	EPI_ISL_19532521	AY.3
S513866	*Odocoileus virginianus*	11/13/2021	SAUNDERS	25.61	338	6x	3.59%		Low coverage
S513869	*Odocoileus virginianus*	11/13/2021	LANCASTER	26.73	33,657	1,211x	92.54%	EPI_ISL_14394050	AY.119
S513882	*Odocoileus virginianus*	11/14/2021	SAUNDERS	27.56	3,115	112x	54.48%		Low coverage
S513890	*Odocoileus virginianus*	11/13/2021	CASS	26.15	502	17x	27.97%		Low coverage
S513891	*Odocoileus virginianus*	11/13/2021	SARPY	25.23	66,041	2,388x	99.36%	EPI_ISL_19534269	AY.46.4
S513895	*Odocoileus virginianus*	11/13/2021	SAUNDERS	27.25	15,102	545x	93.10%	EPI_ISL_14394051	AY.119
S513896	*Odocoileus virginianus*	11/13/2021	SARPY	21.89	67,663	2,446x	99.37%	EPI_ISL_19534270	AY.46.4
S513897	*Odocoileus virginianus*	11/13/2021	SAUNDERS	28.67	7,991	290x	83.03%	EPI_ISL_19532522	AY.46.4
S513899	*Odocoileus virginianus*	11/13/2021	CASS	26.04	475	9x	10.79%		Low coverage
S513901	*Odocoileus virginianus*	11/14/2021	CASS	28.57	33,343	1,207x	96.11%	EPI_ISL_19534271	AY.46.4
S513902	*Odocoileus virginianus*	11/14/2021	CASS	23.98	46,596	1,685x	99.20%	EPI_ISL_19534272	AY.46.4
S513908	*Odocoileus virginianus*	11/13/2021	SAUNDERS	28.62	5,691	199x	69.80%		Low coverage
S513939	*Odocoileus virginianus*	11/13/2021	CASS	27.26	19,889	718x	96.11%	EPI_ISL_14394052	AY.46.4
S513942	*Odocoileus virginianus*	11/13/2021	CASS	28.98	30,317	1,095x	93.06%	EPI_ISL_19534273	AY.46.4
S513948	*Odocoileus virginianus*	11/13/2021	OTOE	24.29	10,234	363x	90.00%	EPI_ISL_19533000	AY.46.4
S513970	*Odocoileus virginianus*	11/13/2021	HALL	25.76	69,892	2,525x	97.95%	EPI_ISL_14394049	AY.46.4
S514021	*Odocoileus virginianus*	11/13/2021	HOWARD	26.54	4,050	142x	46.00%		Low coverage
S514032	*Odocoileus virginianus*	11/15/2021	BUFFALO	29.26	348	7x	3.59%		Low coverage
S514077	*Odocoileus virginianus*	11/20/2021	LINCOLN	24.08	31,019	1,116x	99.36%	EPI_ISL_14394053	AY.100

### 2.5 Genomic sequencing and spatial analysis

Samples were sequenced on the Clear Dx sequencing platform (Clear Labs, CA, United States) using the Clear Dx WGS SARS-CoV-2 assay following manufacturer’s instructions. Input nucleic acid was extracted from SARS-CoV-2 positive samples using MagMAX Pathogen RNA/DNA Kit (Thermo Fisher Scientific, Waltham, Massachusetts, United States), following the manufacturer’s instructions. On the Clear Dx, nucleic acid was amplified using the MIDNIGHT approach (biopipeline BIP-Wv8), libraries were prepared using ONT rapid barcoding kit and sequenced on an R9 flow cell. Demultiplexing of reads, trimming, and assembly of the consensus SARS-CoV-2 genomes are automated using the Clear Labs WGS App.^1^ Genomes with greater than 80% coverage were submitted to GISAID. Assembled genomes were submitted to the web-based version of NextClade^2^ for analysis of substitutions, insertions, and deletions, relative to the SARS-CoV2 reference strain (MN908947) and assignment of Pango lineage (Table 3). The 11 genomes with 90% or greater coverage were submitted to the GISAID AudacityInstant (v5.1.0) using default parameters to find closely related genomes (Supplemental Data Sheet 1). Identified near-neighbors were added to Geneious prime and duplicate sequences were removed and aligned to using MAFFT (v7.490). Phylogeny was reconstructed using IQ-Tree (v.2.2.3) (30) with ModelFinder (31) and visualized using iTol (v6.9.1) (32) and Inkscape (Inkscape Project. v0.92.5^3^). Mutation analysis was visualized using custom python (v.3.12.2) scripts, with libraries including Pandas, Matplotlib, and Seaborn to parse and graph mutation data from NextClade.

ESRI ArcGIS Pro version 3.x (Esri, California, United States) was used to create choropleth and dot density maps to represent the geographic location and density of the sample origins, positive samples for SARS-CoV-2 by RT-PCR and seropositive by sVNT ELISA. Random dot placement was used to obscure the precise location of sample origin and convey the broad spatial distribution of sample test results at multiple scales.

### 2.6 Serology testing procedures

A SARS-CoV-2 surrogate virus neutralization test (sVNT) kit (GenScript; New Jersey, United States) was used to detect neutralizing antibodies against the receptor-binding domain (RBD) of the SARS-CoV-2, isolate BetaCoV/Singapore/2/2020 (Accession ID EPI ISL 406973) (33, 34). According to the manufacturer’s instructions (Version RUO for US 2.0) the percent inhibition cut-off value *<*30% is considered negative and ≥ 30% is considered positive when using cat serum samples. According to Poonsuk et al., a cut-off value of 40% was applied for RLN exudates. The methodological approach was described elsewhere (18).

## 3 Results

### 3.1 Prevalence of SARS-CoV-2 infection in different animal species in Nebraska by RT-PCR

#### 3.1.1 Deer (RLNs, respiratory swab, and Nobuto strip samples)

**Figure 3:**
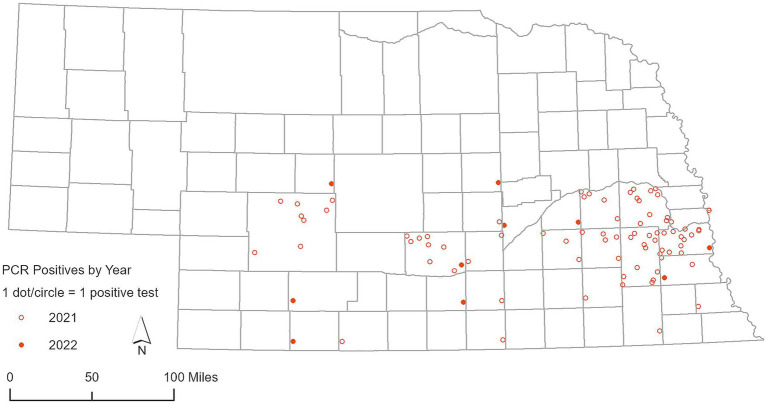
Geographic distribution of deer samples that tested positive for SARS-CoV-2 via RT-PCR in 2021 and 2022.

A total of 1,366 RLNs were analyzed using RT-PCR. SARS-CoV-2 was detected in 79 out of 483 RLN samples from white-tailed deer collected in 2021, representing a detection rate of 16.35%. Viral RNA loads based on RT-PCR cycle threshold (Ct) values of the N1 target was 32 ± 3.3 (mean ± SD), ranging from 23 to 37, and the N2 target was 32 ± 3.7, ranging from 22 to 37. Out of the 43 counties involved in this study, 18 counties had a single positive case of SARS-CoV-2 identified by RT-PCR in white-tailed deer (Figure 3). Many clusters were reported around the Lincoln and Omaha areas and along the Platte River. In 2022, the virus was found in 10 out of 277 RLN samples, which corresponds to a detection rate of 3.61%. Viral RNA loads based on RT-PCR Ct values of the N1 target was 32.50 ± 1.22, ranging from 30.13 to 34.34, and the N2 target was 33.43 ± 1.9, ranging from 29.44 to 36.49, respectively. Specifically, 8 positive cases were identified in white-tailed deer, while 2 were observed in mule deer, marking a notable incidence of infection within these species. No SARS-CoV-2 was detected by RT-PCR in respiratory swab samples (*n* = 159) collected from white-tailed deer and mule deer in 2022. The geographic distribution of deer samples that tested positive for the SARS-CoV-2 RT-PCR among the 49 counties was shown in Figure 3. In parallel, our analysis of 105 Nobuto strips, which were designed to detect viral agents, yielded no positive detections. During our continued surveillance in 2023, the scope was expanded to include 606 RLN samples from 23 counties, which did not detect SARS-CoV-2 in any of these samples.

#### 3.1.2 Peri-domestic feral cats (swabs)

Our comprehensive study conducted in 2022 and 2023 focused on assessing the potential for viral transmission within the feline population. Samples from a total of 623 cats were collected during this study, including 555 cats from the TNR program, 66 respiratory swabs from feral cat carcasses submitted to the NVDC as part of rabies surveillance, and 2 fecal swabs from cats from a household that tested positive for SARS-CoV-2. Despite the extensive nature of this testing effort, all specimens were negative by SARS-CoV-2 RT-PCR.

#### 3.1.3 Other wildlife and zoo animals (swabs)

In an extensive surveillance encompassing other wildlife and zoo animals, our study conducted RT-PCR tests across a diverse array of 1,034 wildlife and zoo animals, as shown in Table 1. This broad-spectrum analysis aimed to identify potential carriers and reservoirs of SARS-CoV-2 beyond the previously reported animal species. Remarkably, the results of this comprehensive testing regimen revealed no detection of SARS-CoV-2 across all species evaluated.

### 3.2 Serological survey of SARS-CoV-2 exposure using surrogate virus neutralization test

#### 3.2.1 Deer (RLNs and Nobuto strip samples)

**Figure 4:**
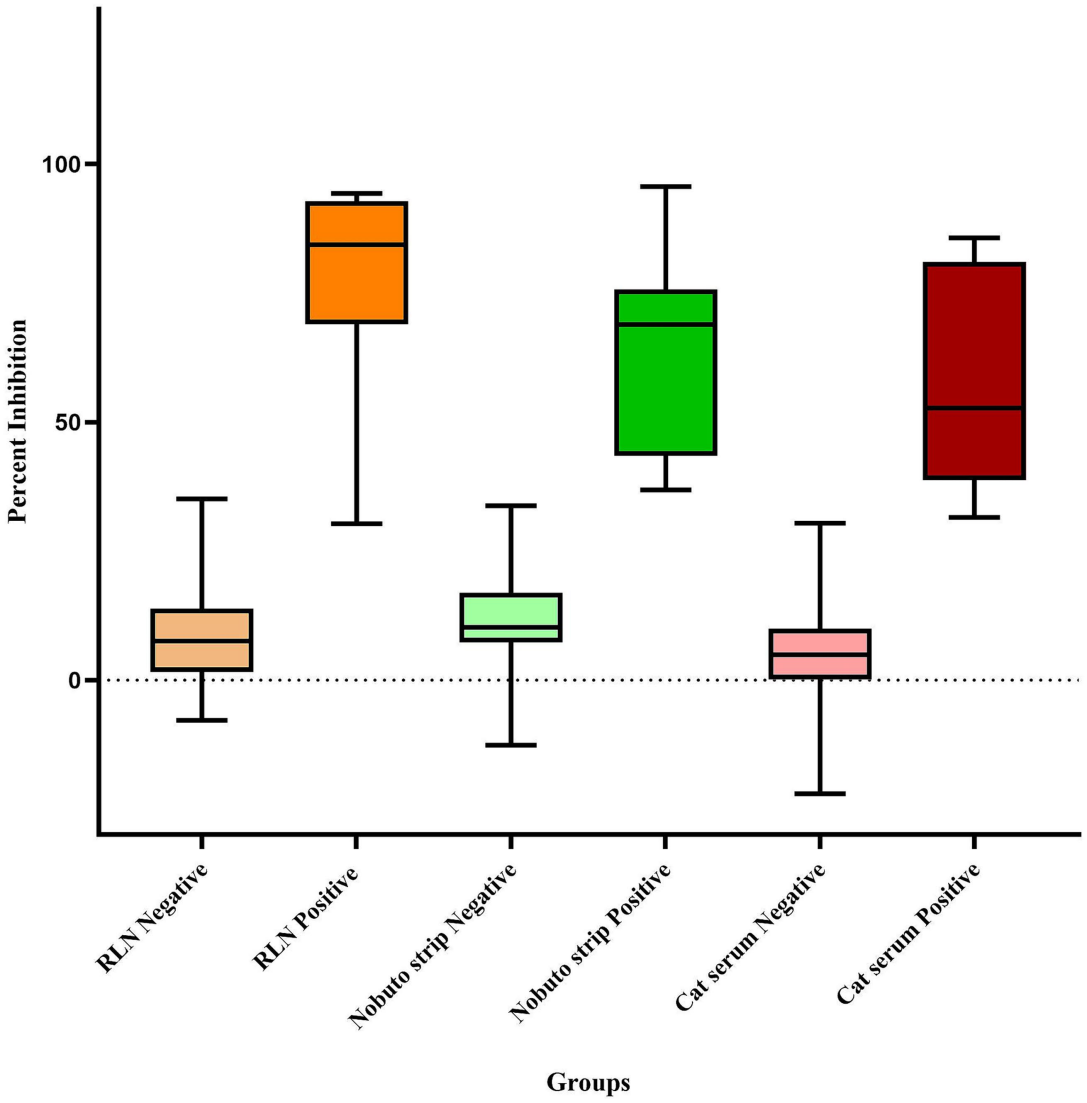
Box and Whisker plot of SARS-CoV-2 serological results for white-tailed deer retropharyngeal lymph nodes (RLN), Nobuto strips, and cat serum samples using the surrogate Virus Neutralization Test (sVNT).

**Figure 5:**
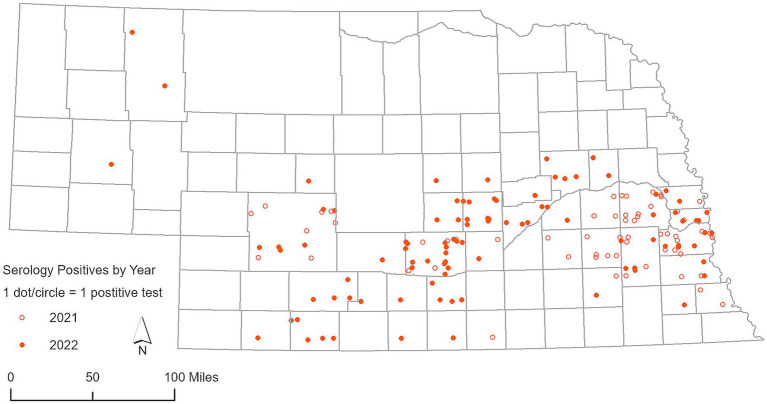
Geographic distribution of deer serum samples and Nobuto test strips that demonstrated exposure to SARS-CoV-2, as detected by surrogate Virus Neutralization Test (sVNT), for years 2021 and 2022.

In 2021, an examination of 91 white-tailed deer RLN exudates showed a significant rate of seropositivity, with 58 samples yielding positive results, indicating a seropositive rate of 63.73% as previously reported by Poonsuk et al. (18). In 2022, we conducted more comprehensive testing by expanding testing efforts to include 237 RLN samples, resulting in 94 positive detections, representing a seropositive rate of 39.66%, indicating a high prevalence of SARS-CoV-2 in white-tailed deer in Nebraska. Percent inhibition of sVNT ELISA ranged from 30.31 to 94.30, with an average of 78.02% (95% CI 63.99–92.05) as shown in Figure 4. The use of Nobuto strips for serological evidence yielded positive results in 15 samples from 76 Nobuto strips. Percent inhibition of sVNT ELISA ranged from 36.78 to 95.65, with an average of 63.83% (95% CI 52.85–74.80) as shown in Figure 4. Of these, 12 samples were corroborated by positive lymph node tests, highlighting a consistent detection of SARS-CoV-2 antibodies from Nobuto strips. Interestingly, 3 cases were solely identified through Nobuto strip testing, two of which had corresponding lymph node samples test negative, and one case where the lymph node was not tested. Combining the results from both lymph nodes and Nobuto strips, we identified a total of 97 positive instances through sVNT ELISA testing, providing a comprehensive view of SARS-CoV-2 seroprevalence among the studied deer populations. No serological testing was undertaken in 2023, marking a pause in our efforts to monitor the seroprevalence of SARS-CoV-2 within these animal populations. Total seropositive samples in 2021 and 2022 are shown in Figure 5.

#### 3.2.2 Peri-domestic cats (serum samples)

**Figure 6:**
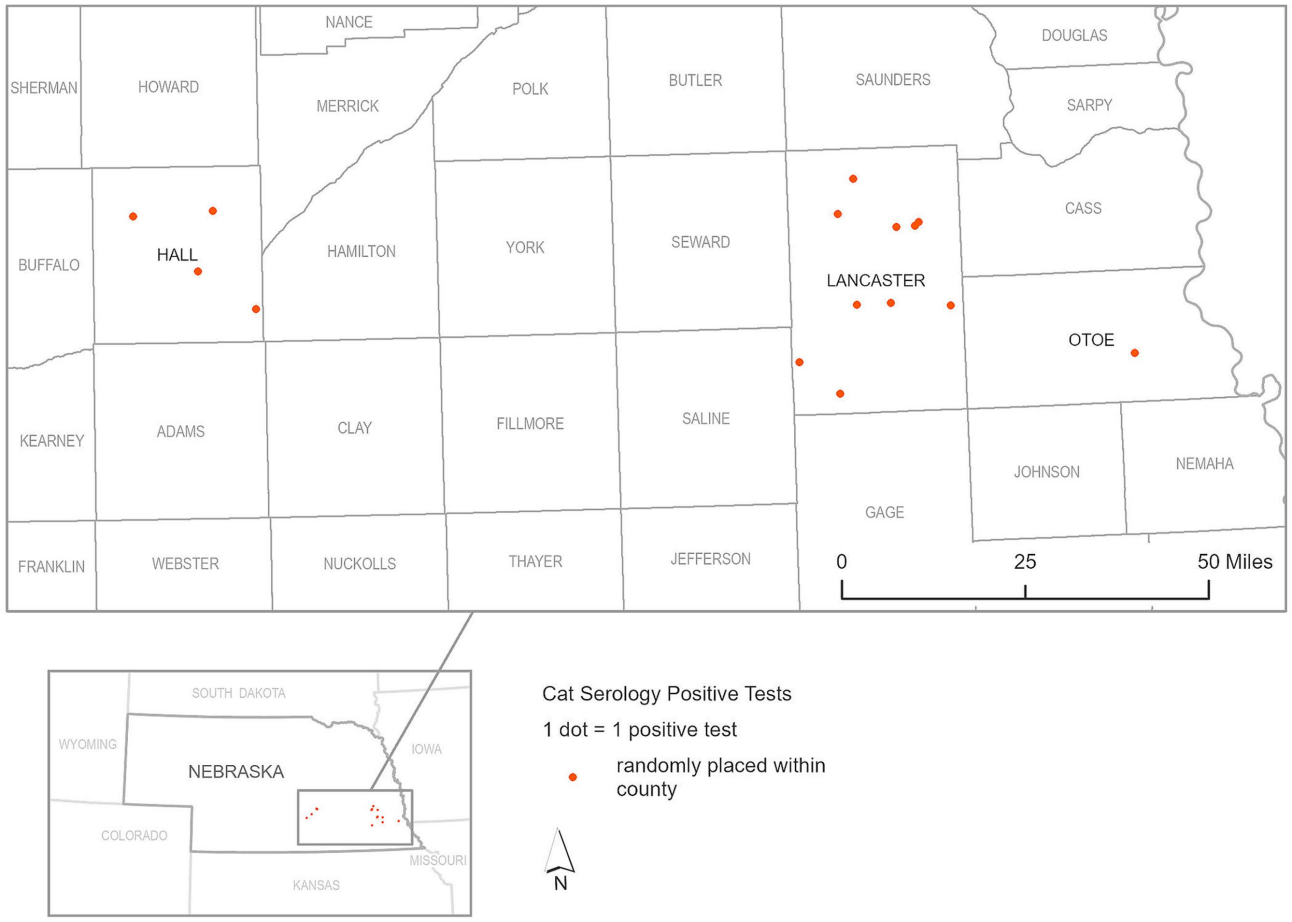
Geographic distribution of seropositive cat serum samples for SARS-CoV-2, as detected by surrogate Virus Neutralization Test (sVNT), during 2022 and 2023.

In our focused serological study of peri-domestic cats participating in TNR programs conducted by the CHS and CNHS during 2022 and 2023, a total of 628 cat serum samples underwent sVNT ELISA testing. Among these, 15 cats tested positive for SARS-CoV-2 indicating a seropositive rate of 2.38%, and percent inhibition ranged from 31.51 to 85.68%, with an average of 57.69% (95% CI 46.20–69.18) as shown in Figure 4, indicating a low but notable the exposure of the virus within this population as shown in Figure 6. The geographical data showed that cats exposed to SARS-CoV-2 were found in three counties including Lancaster, Hall, and Otoe.

### 3.3 Genomic analysis

**Figure 7:**
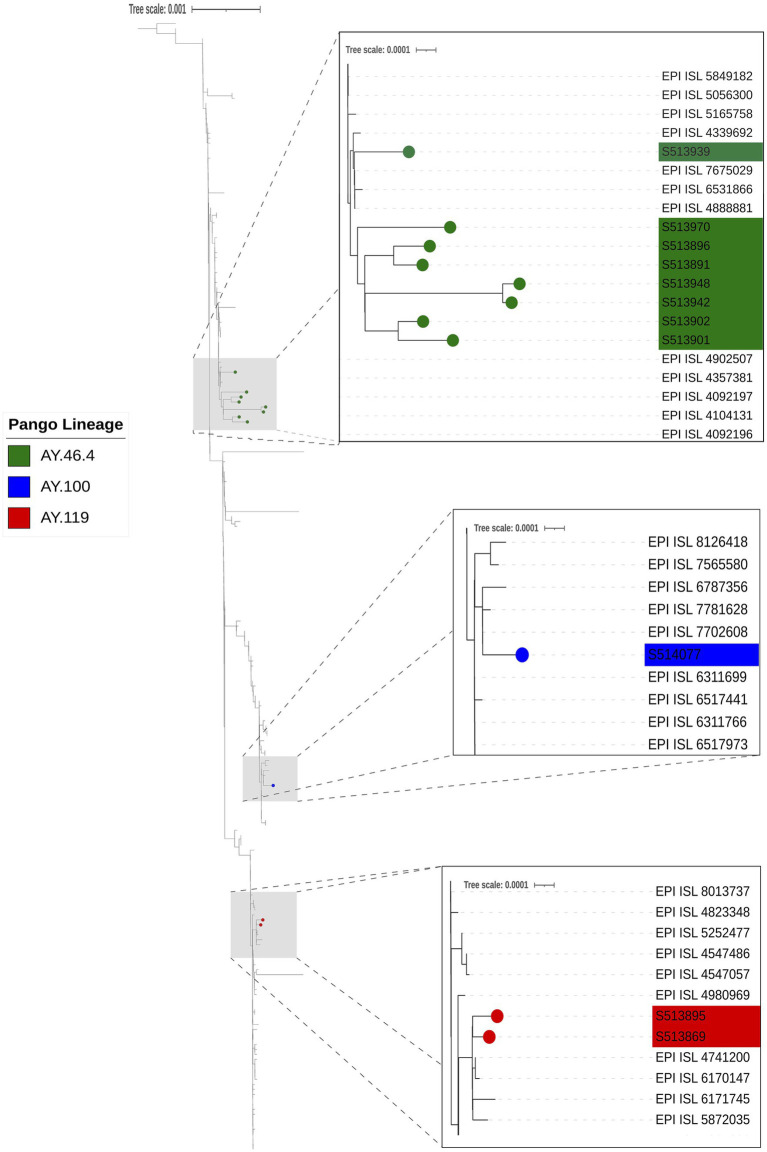
Phylogenetic analysis of SARS-CoV2. The phylogeny was reconstructed using 11 genomes from this study and 215 closely related SARS-CoV2 genomes obtained from GISAID AudacityInstant using IQTree with ModelFinder and visualized in iTOL with Inkscape. The three gray boxes with dashed lines indicate sections of the tree that are zoomed in to visualize SARS-CoV-2 deer genomes from this study clustering with other human SARS-CoV2 genomes (Pango Lineages AY.46.4 [green], AY.100 [blue], and AY.119 [red]), indicating multiple introductions of SARS-CoV2 into the white-tailed deer population in Nebraska.

Whole genome sequencing for SARS-CoV-2 conducted on 20 confirmed positive samples with Ct values of 29 or lower generated genome coverage 20X of over 80% of the genome for 13 samples (Table 3). Sequences were assigned to lineages AY.100 (1), AY.119 (2), AY.3 (1), and AY.46.4 (9). Phylogenetic analysis of the 11 samples with 90% coverage or greater revealed sequences clustered with other human SARS-CoV-2 genomes from Nebraska and that there were multiple introductions of SARS-CoV-2 into the white-tailed deer population in Nebraska (Figure 7). There is no evidence of spillback transmission from white-tailed deer back to humans, as we did not identify any human genomes that were identical or diverging from the white-tailed deer sequences.

**Figure 8:**
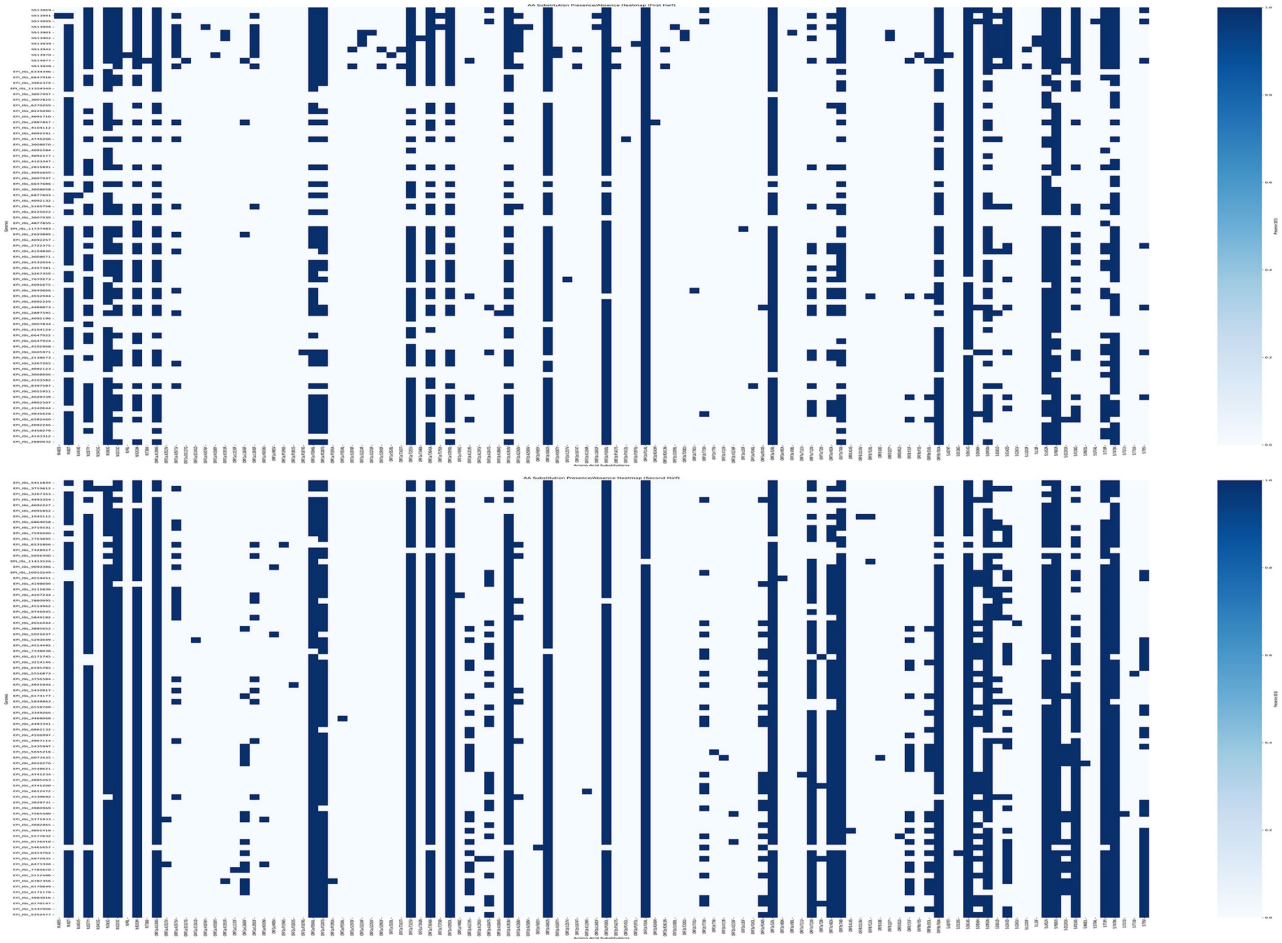
Mutation analysis of SARS-CoV2 Genomes. AA Substitutions were determined by comparing the genomes from white-tailed deer samples from this study and near-neighbors (Supplemental Table S2) to SARS-CoV-2 reference sequence, MN908947. The AA substitutions were plotted for presence (blue) or absence (white) per genome using a custom python script. Thirty mutations were unique to the 11 genomes from this study.

Analyzing AA substitutions across the different white-tailed deer sequence from different lineages of the Delta variant, reveal some specific mutations unique to near-neighbors within the Delta AY lineages (Figure 8). There were 30 unique mutations found in the deer samples compared to the near neighbors (Table 4). However, when mutations were analyzed using outbreak.info mutation tracker (35), only ORF9b:P3S was found to be a unique mutation and found only in one of the deer samples, S513970.

**Table 4:** List of mutations and the number of sequences on GISAID that have the mutation present found in the US or worldwide according to Outbreak.info.

AA mutations	US	Worldwide
ORF1a:T346N	383	982
ORF1a:S528L	1,321	4,138
ORF1a:T1637I	5,390	15,200
ORF1a:D1127G	110	372
ORF1a:S1978F	2,263	7,608
ORF1a:S2224F	5,847	13,585
ORF1a:S2255F	1,360	10,474
ORF1a:H3076Y	6,802	14,275
ORF1a:H3580Y	5,058	12,822
ORF1a:T3750I	130,186	348,529
ORF1a:S3950F	340	1,654
ORF1b:H1087Y	144,138	231,592
ORF1b:S1089L	5,633	13,760
ORF1b:P1427S	9,913	32,442
ORF1b:I1674T	982	5,257
ORF1b:L1681F	7,943	34,206
ORF1b:P1975L	386	1,435
ORF1b:T2081I	237	602
ORF1b:A2589V	417	1,851
ORF1b:R2613N	13,824	46,205
S:L18F	41,785	247,574
S:S704L	247,647	340,078
S:A879T	531	1,356
S:L1203F	932	3,167
ORF3a:V88L	2,398	11,236
M:A85S	2,097	4,010
ORF8:Q27*	246,084	1,170,076
ORF9b:P3S	0	0
N:P6L	3,255	13,147
N:T366I	8,652	30,499

## 4 Discussion

This large surveillance study involved extensive field sampling and testing of 5,794 samples from 44 animal species, representing 90 out of 93 counties in Nebraska. The primary findings included high rates of detection (16.35%) (*n* = 483) geographic dispersion (18 out of 43 counties), and the subsequent disappearance of virus in 2023 (*n* = 606 samples). This indicates potentially higher levels of population immunity, leading to lower to undetectable levels of virus detection in deer by 2023. Given the variation in geographic distribution of sampled areas across the years, some of the variation in prevalence of the virus presence may be attributable to this factor. The lack of detection of SARS-CoV-2 on any sample but RLN indicates potential importance of lymphoid tissues for virus surveillance in deer. Serological surveillance can be used to determine exposure in animals without active infections. Seropositivity rates also support widespread prior circulation of SARS-CoV-2 with rates found to be 63.73% (*n* = 91) and 39.66% (*n* = 237) in 2021 and 2022, respectively. The Nobuto strip was also successfully used for serological surveillance (20) and provided 3 additional positive samples beyond those detected in RLN exudates. These findings emphasize the utility of diverse testing methods in capturing a broader picture of SARS-CoV-2 exposure. Both RT-PCR and serology results highlighted that deer are a potential reservoir of SARS-CoV-2, as previously described in other states (13–16, 20).These RLN found positive by RT-PCR were collected during the hunting season of November–December 2021, which coincided with a spike in human SARS-CoV-2 infections in Nebraska, with the highest number of cases peaking in January 2022 (36). This was particularly notable in the most populated counties such as Douglas and Lancaster. These counties experienced high levels of virus transmission among humans, which may be related to the higher virus detection levels and seropositivity in samples from the eastern part of Nebraska during this period. This pattern suggests that the timing of sample collection and the prevalence of human infections may influence the observed distribution of positive samples.

SARS-CoV-2 has been detected in various captive animals, including tigers, lions, snow leopards, cougars, lynxes, fishing cats, binturongs, hyenas, otters, coatimundis, hippos, and gorillas (37, 38). However, other wildlife species included in this surveillance did not show virus detection, even with serial sampling conducted over periods of widespread virus circulation in humans. This information aligns with our understanding of SARS-CoV-2 animal reservoirs and supports findings from other studies that white-tailed deer are susceptible and have high levels of exposures. Seroprevalence of peri-domestic cats showed a 2.38% (*n* = 628) seropositive rate in 2022, with no detections by RT-PCR, indicating low rates of previous infection and low levels of potential exposure. Although it remains unclear why peri-domestic cats have such lower levels of exposures compared to white-tailed deer, even though they are roaming in nature and have interaction with other cats, this fits with the existing literature. In seroprevalence studies with cats that were not associated with SARS-CoV-2 detections or COVID-19 positive households had positivity rates ranging from 0.7–3.5% (39), indicating there did not appear to have been widespread circulation of SARS-CoV-2 in peri-domestic or other domestic cats. Experimental infectious of cats and transmissibility studies demonstrate cat-to-cat transmission, however, passaging in cats decreased transmissibility and pathogenicity (11). There have been numerous cases of human-to-cat transmission, but no evidence supporting cat-to-human transmission (40, 41), however there remains significant gaps in understanding the role cats play in natural infection, however evidence suggests that cats have the potential as spillover hosts, but there lacks evidence that they have the capacity to widely transmit among other cats or serve as a reservoir host.

Our sequencing and phylogenetic analysis of SARS-CoV-2 samples from white-tailed deer in Nebraska revealed the presence of multiple Delta variant lineages. This observation aligns with broader epidemiological trends in human populations during the same timeframe when this variant was prevalent, underscoring the potential role of deer and other animals in sustaining and potentially spreading variants of concern like Delta (16). Even though our study found no evidence of transmission from animals to humans, other studies have shown evidence of bidirectional transmission between humans and wildlife (15–17, 42–44). The identification of multiple lineages of the Delta variant (AY.100, AY.119, AY.3 and AY.46.4) in the Nebraska deer population suggests that SARS-CoV-2 is spillover over from humans to animals on multiple occasions. The direct relationships between virus circulating in human populations and deer, and how interactions between those populations may have affected virus transmission remain unclear. The Delta variant detection in humans in Nebraska in November 2021, as recorded in GISAID, was widespread both in Nebraska and globally during November–December of 2021 and early 2022, which coincided with the hunting season and sample collection periods. This widespread detection in both populations supports the evidence for SARS-CoV-2 introduction to the deer population in Nebraska. Indicating that surveillance is crucial to monitor the introduction and spread of new variants within wildlife populations from humans or otherwise.

The challenges encountered in sequencing for a significant proportion of the samples highlight the need for improved methodologies in wildlife genomic studies. Of the 20 selected low Ct SARS-CoV-2 samples, only 13 genomes passed the QC criteria we set (80% coverage) for GISAID submission. To put this in context, based off of previously sequenced human clinical, we expected at least 80% or higher coverage from all the selected deer samples. This discrepancy demonstrates the challenges in obtaining high-quality genomic data from wildlife samples, which can be affected by factors such as sample degradation, autolysis, and contamination. Factors such as sample quality, collection methods, and storage conditions play a critical role in the success of sequencing efforts. The method of sequencing should also be taken into consideration and use of smaller amplicon size could help to negate issues with degradation. Future studies should focus on optimizing these variables to increase the yield of high-quality genomic data from wildlife samples. Additionally, the variation in sample types by species and management strategies made it difficult to optimize a single sample type for all. Also, the geographic distribution of sampling sites was not evenly distributed which may affect our ability to fully understand disease distribution.

In conclusion, the detection of SARS-CoV-2 in white-tailed deer has potential significant implications for public health and wildlife management. Deer populations could serve as reservoirs for the virus, enabling sustained transmission and the potential emergence of new variants. This is particularly concerning for variants that have demonstrated increased transmissibility, virulence, or immune escape capabilities. However, the absence of detectable virus in 2023 may indicate that the deer largely developed population-level immunity and the virus may no longer be widely circulating in these populations. Additionally, the low levels of seropositivity observed in feral or unmanaged cat populations indicate that they may not be serving as a significant reservoir host to any large degree, at least in the population evaluated. However, the detection of at least some seropositivity indicates that they may have the potential to become infected in field settings. This highlights the need for comprehensive assessment of virus circulation that includes both antibody based tests that can detect previous exposure, along with virus detection tests to allow for rapid detection of circulating virus in multiple populations. The use of host agnostic tests (sVNT and RT-PCR) allow for widespread surveillance in diverse animal species to promote a more comprehensive understand of virus dynamics.

Our findings underscore the importance of implementing comprehensive surveillance programs that include both domestic animals and wildlife. Such programs are essential for early detection of spillover events for SARS-CoV-2 and potentially other viruses, and evaluating for potential spread across different species. Enhanced surveillance efforts should be coupled with genomic sequencing to track the evolution of the virus and to inform public health strategies aimed at mitigating the risk of zoonotic transmission. Additional longitudinal studies may provide further information on virus transmission, spread, and sequence variation among wildlife populations such as deer, and would further help understand the mechanisms of maintenance and transmission in wildlife, and potential One Health implications thereof.

## Data Availability

The datasets presented in this study can be found in online repositories. The names of the repository/repositories and accession number(s) can be found in the article/Supplementary material.

## Footnotes

^1^ https://wgs.app.clearlabs.com/

^2^ clades.nextstrain.org

^3^ https://inkscape.org
