# Supplementary material for: SARS-CoV-2 surveillance and detection in wild, captive, and domesticated animals in Nebraska: 2021–2023
Source: Front Vet Sci. 2025 Jan 3;11:1496207. doi: 10.3389/fvets.2024.1496207 (PMC11739072; doi:10.3389/fvets.2024.1496207)
Supplement: Supplementary file 1 [file Data_Sheet_1.PDF]

SUPPLEMENTAL TABLE

Data Availability

GISAID Identifier: EPI_SET_241110hq
doi: 10.55876/gis8.241110hq

All genome sequences and associated metadata in this dataset are published in GISAID's EpiCoV database. To view the contributors of each individual sequence with details such as accession number, Virus name, Collection date, Originating Lab and Submitting Lab and the list of Authors, visit 10.55876/gis8.241110hq

Data Snapshot

- EPI_SET_241110hq is composed of 215 individual genome sequences.
- The collection dates range from 2021-03-30 to 2021-12-21;
- Data were collected in 11 countries and territories;
- All sequences in this dataset are compared relative to hCoV-19/Wuhan/WIV04/2019 (WIV04), the official reference sequence employed by GISAID (EPI_ISL_402124). Learn more at https://gisaid.org/WIV04.
