# Supplementary material for: SARS-CoV-2 surveillance and detection in wild, captive, and domesticated animals in Nebraska: 2021–2023
Source: Front Vet Sci. 2025 Jan 3;11:1496207. doi: 10.3389/fvets.2024.1496207 (PMC11739072; doi:10.3389/fvets.2024.1496207)
Supplement: Supplementary file 2 [file Table_1.DOCX]

**Supplementary Table 1.**

The county's location of deer sample collection from 2021 to 2023 as part of a statewide Chronic Wasting Disease (CWD) surveillance program by the Nebraska Game and Parks Commission.

| **Numbers** | **2021** | **2022** | **2023** |
| --- | --- | --- | --- |
| 1 | Adams | Antelope | Antelope |
| 2 | Buffalo | Banner | Blaine |
| 3 | Butler | Boone | Boone |
| 4 | Cass | Buffalo | Boyd |
| 5 | Clay | Butler | Brown |
| 6 | Custer | Cass | Cherry |
| 7 | Dawson | Cherry | Custer |
| 8 | Dodge | Cheyenne | Garfield |
| 9 | Douglas | Colfax | Grant |
| 10 | Fillmore | Custer | Greeley |
| 11 | Franklin | Dawes | Holt |
| 12 | Frontier | Dawson | Hooker |
| 13 | Furnas | Douglas | Keya Paha |
| 14 | Gage | Franklin | Logan |
| 15 | Garden | Frontier | Loup |
| 16 | Gosper | Gage | McPherson |
| 17 | Hall | Garden | Rock |
| 18 | Harlan | Gosper | Sheridan |
| 19 | Howard | Greeley | Sherman |
| 20 | Jefferson | Hall | Thomas |
| 21 | Johnson | Harlan | Valley |
| 22 | Kearney | Hayes | Wheeler |
| 23 | Keith | Hitchcock |  |
| 24 | Lancaster | Howard |  |
| 25 | Lincoln | Johnson |  |
| 26 | Logan | Kearney |  |
| 27 | Merrick | Keith |  |
| 28 | Nemaha | Lancaster |  |
| 29 | Nuckolls | Lincoln |  |
| 30 | Otoe | Logan |  |
| 31 | Pawnee | Merrick |  |
| 32 | Phelps | Morrill |  |
| 33 | Platte | Nance |  |
| 34 | Polk | Otoe |  |
| 35 | Richardson | Phelps |  |
| 36 | Saline | Platte |  |
| 37 | Sarpy | Polk |  |
| 38 | Saunders | Red Willow |  |
| 39 | Seward | Saline |  |
| 40 | Thayer | Sarpy |  |
| 41 | Washington | Saunders |  |
| 42 | Webster | Scotts Bluff |  |
| 43 | York | Seward |  |
| 44 |  | Sheridan |  |
| 45 |  | Sherman |  |
| 46 |  | Sioux |  |
| 47 |  | Thomas |  |
| 48 |  | Thurston |  |
| 49 |  | Valley |  |
